# On the automated translational execution of the action language for foundational UML

**DOI:** 10.1007/s10270-016-0556-7

**Published:** 2016-09-26

**Authors:** Federico Ciccozzi

**Affiliations:** Department of Innovation, Design, and Engineering (IDT), MRTC, Mälardalen University, 72123 P.O. Box 883, Västerås, Sweden

**Keywords:** Model-driven engineering, Translational execution, Code generation, UML, ALF

## Abstract

To manage the rapidly growing complexity of software development, abstraction and automation have been recognised as powerful means. Among the techniques pushing for them, model-driven engineering has gained increasing attention from industry for, among others, the possibility to automatically generate code from models. To generate fully executable code, models should describe complex behaviours. While pragmatically this is achieved by employing programming languages for defining actions within models, the abstraction gap between modelling and programming languages can undermine consistency between models and code as well as analysability and reusability of models. In light of this, model-aware action languages should be preferred. This is the case of the Action Language for Foundational UML (ALF). In this paper, we provide a solution for the fully automated *translational execution* of ALF towards C++. Additionally, we give an insight on how to simplify the transition from the use of programming languages for modelling fine-grained behaviours to model-aware action languages in industrial MDE. The solution presented in this paper has been assessed on industrial applications to verify its applicability to complex systems as well as its scalability.

## Introduction

Software pervades our everyday life in many ways and its complexity is continuously increasing, thus demanding both methodological and technical enhancements of existing engineering approaches. Already from the early 50s, when software became too complex to be defined in terms of machine languages by hand, the need for simplification by *abstraction* led to the creation of a plethora of programming languages [51]. During the 90s, it became clear that the very fast growth of software’s complexity would lead to the need of defining even more powerful and flexible development approaches [20]. Once again the need of further abstraction arose [29]. On one hand, abstraction can effectively help in mitigating software’s complexity. On the other hand, it introduces additional artefacts and development phases (e.g. design, transition from design to implementation) and thereby the need of ensuring consistency among them. That is to say, while taming the complexity of the software to be developed, abstraction may make the engineering process more intricate [5]. A common way to mitigate the complexity of an engineering process, especially when dealing with software systems, is boosting *automation* in the various engineering phases.

In the profusion of software engineering techniques advocating abstraction and automation as compelling needs for effective development, model-driven engineering (MDE) has stood out and got a foothold as a promising way to (i) tackle the difficulty of third-generation languages to effectively mitigate software’s complexity and express domain-specific concepts, and (ii) alleviate the complexity of the engineering process by providing means for automation. To manage software’s complexity, MDE aims at promoting models as primary artefacts in the development.

MDE advocates automation by means of transformations between models at several development stages for model-based analysis, model simulation, code generation and even back-propagation from code to models [10], to mention a few [44]. Generally, in order for the adoption of MDE to be profitable from an industrial point of view, it has been empirically assessed that automated code generation cannot be neglected [21]. One of the crucial characteristics for a modelling language to suffice as input for full-fledged code generation is the ability to provide means for specifying fine-grained behaviours. Most often, this is done by inserting code written in common programming languages (e.g. C++, Java) as behavioural descriptions in the model.

On one hand, this represents a pragmatic way to address the problem in industrial settings since it enables the reuse of legacy models with fine-grained behaviours defined through programming languages. On the other hand, this practice can bring more drawbacks than benefits when it comes to consistency, analysability, reusability, just to mention a few, as explained in the remainder of the paper. These drawbacks can be tamed through promoting the use of model-aware action languages, based on the modelling languages themselves, as the preferred way for defining fine-grained behaviours when modelling a software system. This is the case of the Action Language for Foundational UML (ALF) [36] defined by the Object Management Group (OMG) to act as the surface notation for specifying executable behaviours within a wider model that is primarily represented using the graphical notations of the Unified Modelling Language (UML)^1^. ALF naturally leverages the UML metamodelling elements and thereby can boost consistency-by-construction and ease model-based activities (e.g. analysis [12], simulation [14]).

In this paper, we provide a first of its kind solution for the automatic *translational execution* (see Sect. 2) of ALF to C++. While in our preliminary results [9] we introduced the idea and showed that such a translation was possible, in this contribution we provide a fully functioning solution for translating (i) all the ALF concepts needed for defining complex behaviours and (ii) a portion of those needed for structural definitions. Moreover, we provide a solution for both memory management and type deduction. A detailed description of the contribution in relation to our preliminary results is provided in Sect. 3.

Since the use of programming languages within models is rooted in UML-based industrial MDE, we do not advocate a radical and sudden shift to ALF which would not be feasible in industry. To support this, in the paper we indicate how our translator from ALF to C++ can be exploited as a complement to existing MDE processes that generate executable C++ from UML with C++ as action code. Doing so, legacy models (or parts of them) can be reused and the adoption of completely UML-compliant model-driven approach by designing new models (or parts of them) entirely using UML and ALF can be gradual. The final goal would be to model using UML (and profiles) and ALF only.

The translation mappings have been tested through transformation unit testing [50], while functional correctness of the generated C++ code has been assessed through monitoring and logging routines on instrumented code. Among the applications used for validation and evaluation purposes, the self-orienting carrier robot system is used in the paper as running example for showing the translation process. Moreover, the Asynchronous Transfer Mode (ATM) Adaptation Layer 2 (AAL2) subsystem, defined within Ericsson Nikola Tesla in Zagreb (Croatia) under the supervision of Ericsson AB in Kista (Sweden), has been exploited also to assess scalability and applicability to real industrial systems.

The remainder of this paper is organised as follows. In Sect. 2, we describe the core concepts that set the scope of our problem and solution. A snapshot of the related works, the details of the presented contribution and how it is meant to advance the current state of the art and practice are presented in Sect. 3. In Sect. 4, we provide a high-level description of the solution, while the complete list of mappings from ALF syntax elements to C++ is given in Sect. 5. In Sect. 6, we show the application of the transformation process to a running example. Details on validation and evaluation of the solution are described in Sect. 7, while Sect. 8 proposes a discussion of core aspects related to problem and solution. The paper is concluded with a short summary of the presented work as well as possible and planned future enhancements in Sect. 9.

## ALF: why and how?

The central concept of MDE is the *model*, an abstraction of a real problem conforming to a metamodel, which describes the set of concepts and wellformedness rules that a model shall follow. In the abundance of general-purpose and domain-specific modelling languages, UML has emerged and established itself as de facto standard in industrial model-based development of software systems [22], and, more generally, empirically proven to be the most widely used architectural description language [31]. The propensity for adopting UML is partially motivated by its versatility, which enables (i) its usage as general-purpose language and (ii) the possibility to customise it through the so-called profiling mechanisms [1] to give it domain specificity.

Although helping in raising the level of abstraction, initially UML was not sufficiently powerful to describe executable programs as traditional third-generation programming languages were, and therefore it had to rely on them to achieve executable artefacts. This was, and still is, done either by employing these languages for describing behaviours within the models or by producing structural code skeletons to be completed by hand with specific behaviours. With UML 1.5, an action semantics for UML was introduced. Moreover, the standardisation of (i) the Foundational Subset For Executable UML Models (fUML), which gives a precise execution semantics to a subset of UML limited to composite structures, classes and activities (application models designed with fUML are executable by definition) [48], and (ii) a textual action language, ALF, to express complex execution behaviours, has made UML a full-fledged implementation quality language [45].

ALF is a textual surface representation for UML modelling elements, whose execution semantics is given by mapping ALF’s concrete syntax to the abstract syntax of fUML. The primary goal of ALF is to act as the surface notation for specifying executable behaviours within a model represented using the usual graphical notations of UML. Additionally, ALF comes with an extended notation that can be used to represent structural modelling elements too. That is to say, it is possible to describe a UML model entirely using ALF. Anyhow, ALF’s syntax covers only the limited subset of UML structural modelling concepts accounted in fUML. While ALF maps to fUML in order to provide its execution semantics, its use is not limited to the context of models conforming to the fUML subset. ALF has been designed with Java-like syntax, but incorporates UML’s textual syntax too.

In Listing 1, we can see an example of ALF code where ALF concepts (expressions and statements) are intertwined with UML concepts (classes, attributes, methods—see UML model in “Appendix A”). 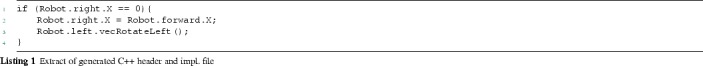


Nonetheless, approaches employing programming languages for action code still dominate industrial MDE for pragmatic reasons; anyhow, the many issues brought about by this practice are undeniable. An example: how to maintain, or even simply check, consistency at modelling level when the abstraction gap between modelling and programming languages hinders action code from being naturally aware of surrounding modelling concepts? Another issue is represented by the fact that, by using programming languages for defining action code, modellers may infer assumptions on the target language or platform (e.g. memory management, parallelism, communication mechanisms), which undermine reusability of models, especially if the target language or platform change. These are crucial reasons for which the use of model-aware action languages like ALF, based on the modelling languages themselves, should be preferred to programming languages when modelling fine-grained behaviours.

With the standardisation of ALF, we noticed an increasing industrial interest in gradually moving towards legitimate action languages. Anyhow, such an adoption would not be painless nor immediate since the use of programming languages within models is rooted in UML-based industrial MDE. This is due to the fact that pragmatism and attention to costs, core priorities in industry, are in harmony with maximised reuse (in this case reuse of legacy models). This is why we provide a solution which can support and boost this adoption process by giving the possibility to exploit ALF as a complement to existing UML-based code generators. This means that MDE processes generating executable C++ from UML with C++ as action code could progressively move towards a completely UML-compliant model-driven approach by designing new models (or new parts of existing ones) entirely using UML and ALF.

According to its specification, ALF has three prescribed ways to achieve *semantic conformance*, meaning how execution semantics is implemented, summarised as follows:

- **Interpretive execution:** ALF is directly interpreted and executed;
- **Compilative execution:** ALF is translated into a UML model conforming to the fUML subset and executed on the actual target platform according to the semantics specified in the fUML specification;
- **Translational execution:** ALF, as well as any surrounding UML concept in the model, is translated into an executable for a non-UML target platform and executed on it.

We focus on the *translational execution* for producing C++ code. The proposed solution has been developed within the industrial consortium specifically devoted to Papyrus [17] that has recently been approved by the Eclipse Foundation^2^, in tight collaboration with Ericsson. Technically, the solution is developed as a set of plug-ins on top of Papyrus.

Automation in MDE is usually achieved through model transformations, which provide the links between domain abstractions, including those between models and source code, and represent a fundamental aspect in automating development. A model transformation converts source models into target artefacts (i.e. models or text) related to the same system by means of a transformation specification [13]. We exploit model-to-text transformations, which we define in terms of the Xtend^3^ language. Xtend is regarded as a flexible dialect of Java, which is transparently compiled into readable Java 5 compatible source code and can seamlessly exploit existing Java libraries. We used Xtend since, compiling to Java and therefore interpreted by the JVM, it resulted in being much faster of other transformation languages.

An alternative to model transformations could have been the exploitation of compile-time metaprogramming by defining macros for ALF (an example for the Scala language is described in [6]). Although a valuable add-on to the ALF language itself, macros would not allow us to treat ALF (and surrounding UML) as models within EMF and Papyrus, which is one of the core prerequisites for our solution to be fully compliant with the latest official implementation of ALF.

It is noteworthy to clarify that in this work we focus on the problems related to automated generation of 100% code from models. We aim at generating full-fledged executable code from UML, and action code written in ALF, which is comparable to what is currently generated exploiting UML, and C++ for action code; thereby, a comparison with handwritten and manually tuned optimised code is out of scope.

## Advancing the state of the art and practice

In this section we provide a snapshot of the state of the art and practice and we describe how this paper is intended to contribute to it.

### Related work

Initially, UML was not sufficiently powerful and precise to produce executable code as traditional third-generation programming languages were. For this reason, UML had to rely on either existing programming languages or adapted dialects for being able to generate full-fledged code. As soon as UML embarked on life, several solutions for translation execution of UML models were proposed and, some of them, commercialised. Many (e.g. [15]) have exploited UML and its profiles to generate code skeletons, but did not focus on generating full-fledged code. Commercial tools such as Enterprise Architect [47], IBM Rational Rhapsody [23] and IBM Rational Software Architect [24] have historically exploited programming languages to define behaviours within UML models and generate full-fledged code. As mentioned previously in the paper, this practice brings a set of drawbacks, such as intricate model validation, analysis and consistency checking. Moreover, by expressing action code through programming languages, the developer infers assumptions on the target platform (e.g. memory management, parallelism, communication mechanism), which may hinder the generation of code for different targets from the same input models.

Concerning proper action languages, some of the existing ones are inspired by the action semantics of UML, but none of them is conformant to the standardised execution semantics defined through fUML. Chronologically, the Shlaer–Mellor Action Language (SMALL) [39] was the first of its kind and was specified to exploit a data flow-based execution similarly to fUML; the language was never implemented. In the context of the object-oriented analysis (OOA) tool for executable UML, the Action Specification Language (ASL) [28] was defined and it represented a fairly capable action language at the time. The OOA tool provided some limited code generation features, which were further enhanced with its descendent, MentorGraphics’s BridgePoint [32]. This tool provided a powerful action language, namely the Object Action Language (OAL) [33], which can be seen as a proprietary dialect of the predecessor of ALF, the UML Action Language (UAL). UAL was adopted and customised [30] by IBM too, as part of the their Rational Software Architect tool [24].

Another proprietary language is the Platform-independent Action Language (PAL), which was not based on the formal execution semantics of UML. The PathMATE tool exploited it to provide assisted code generation based on models and marking [37]. PAL was extended with concepts from the Object Constraint Language (OCL)^4^ by Motogna et al. [35]. OCL was exploited again by Jiang et al. [26], who presented the OCL4X action language, where OCL was enriched with meta-actions for changing the system state. $+$CAL [38] was presented as an action language based on the action semantics of UML, but, focusing specifically on distributed real-time embedded systems, it did not provide the needed degree of generality that should be peculiar of an action language. A Java-inspired attempt is represented by the Action Language for Business Logic (ABL) [19]. The action language is meant to be converted to actions defined with the action semantics of UML, but includes elements that are not part of it. An investigation and evaluation of some UML action languages is provided in [2]

Since its standardisation, UML has triggered several initiatives for defining actions, mostly towards model simulation and code generation. This has been achieved through the exploitation of programming languages, the customisation of the UML action semantics, etc. Nonetheless, to the best of our knowledge, none of the approaches documented in the literature provide a solution for the automatic generation of full-fledged code from the de facto standard action language for UML, ALF. One could argue that modern object-oriented languages, such as Scala^5^ and Xtend, for which code generators exist [4, 41], might be employed instead of ALF. Although similar from an expressive power perspective, none of those languages exhibit the awareness of surrounding model elements that ALF provides.

In the literature, interpretive approaches can be found too. Among them, none provides a solution for the execution of UML models on the actual target platform, but rather focus on middleware-based execution for simulation [49] and model-based analysis [3]. Moreover, only one of the approaches [49] employs ALF. While very useful at design level for early analysis, interpretive solutions, usually based on resource-demanding middleware (or even on the modelling environment itself), need to be complemented with a generative approach producing executable code to be run on resource-constrained targets (e.g. embedded systems).

The literature offers only three compilative approaches, with no continuity in time (they are spread over a 10-year span) [42, 43, 46]. In [42], the authors propose a front end for GCC and enhance dead code elimination optimisation and block merging. The other two solutions are hybrid, since they compile UML models to a format that is interpreted by ad hoc virtual machines. Moreover, *none of the approaches entail ALF*. The three approaches leverage very limited subsets of UML and exploit only graphical behavioural diagrams (e.g. state machines and activities).

Since our goal was to provide a non-breaking solution to boost the adoption of ALF in UML-based MDE even in industrial settings, we chose to go for translational execution. This is justified by the state of the practice in industry where executable code in terms of a third-generation programming language (e.g. C++) is crucial to be able to reuse existing domain-specific runtime layers and optimised compilers.

### Paper contributions

In this paper, we provide *the first of its kind solution for the fully automated translational execution of ALF*. To the best of our knowledge, there is no documented attempt in the literature addressing the problem of generating full-fledged executable code from (UML and) ALF, except for our preliminary effort in this direction [9]. More specifically, we provide the following.

**Translation of behaviours.** We provide translation of ALF concepts within the syntactical *minimum conformance* (as described in the ALF specification), that is to say the subset of ALF that is used for writing textual action language snippets as behaviours within a larger graphical UML model and that includes all the capabilities available in a traditional, procedural programming language. In our previous work [9], we provided a solution limited to a small subset of the minimum conformance considering a first implementation (metamodel in Papyrus) of ALF which did not fully reflect the specification. In this solution, we employ the implementation based on the latest ALF specification (v1.0.1); this resulted in a completely new transformation process, which does not leverage anything of its limited predecessor. As aforementioned, fine-grained behaviours have historically been modelled exploiting programming languages. Since a breaking solution “forcing” the sudden adoption of ALF would not be feasible in industrial settings, we show how ALF and our translational execution can be exploited as a complement to existing code generators; the motivations behind such a feature were introduced in [8]. More specifically, we show how to generate C++ from models whose structure is defined through UML and fine-grained behaviours by action code written in ALF. To the best of our knowledge, there is no documented attempt in the translation of ALF to any general-purpose programming language, including C++.

**Translation of units.** We provide a translation of part of the concepts, addressed as ALF *units*, that are used to textually describe structural portions of a UML model (within the fUML subset). More precisely, we provide support for the translation of: namespace, package, class (passive), operation, property. By providing this option, we allow the developer to, besides defining actions within a graphical UML model, even define the structural parts of the model in terms of ALF to get corresponding executable C++ generated entirely from an ALF model. In our previous work, we did not address ALF units. Solutions for the translation of these same structural portions from UML and its profiles (not ALF) to general-purpose programming languages exist [7]. However, these solutions are usually based on the UML metamodel, while we provide a solution tailored for the ALF metamodel in order to enable the possibility to describe a fully functional model entirely leveraging ALF and its abstract syntax.

**Memory management.** ALF does not enforce a specific memory management mechanisms, providing the possibility to create and destroy objects explicitly, or to constrain an object’s life cycle to the so-called execution locus. Since there was not a straightforward way to bridge memory management mechanisms in ALF and C++, we investigated the set of possible solutions for managing memory in C++ (i.e. garbage collection, smart pointers, manual memory management) and opted for memory management based on *smart pointers* 6. Although there is a noteworthy body of the literature addressing issues related to memory management when building compilers, none deals explicitly with UML’s action languages in general nor their translation in particular. In our previous work, we provided hard-coded manual memory management relying on static object instantiation only.

**Type deduction.** We provide a type deduction mechanism as part of the transformation process. This mechanism is exploited for simplifying the type setting of objects by deducing at a glance types without having to continuously navigate the ALF syntax tree. For instance, in ALF, access to members achieved through a dot operator (‘.’) can result, in C++, into dot, arrow (‘->’) or semicolon (‘::’) operators depending on the type of objects as well as the memory management mechanism. In order to correctly generate access to members, we need type deduction mechanisms. More specifically, the core functionalities provided by the type scope mechanism are four: (1) create, edit and retrieve scopes following their hierarchical structure in the ALF blocks, (2) create, overwrite and retrieve sub-scopes associated with an ALF element, (3) search of names already declared within current scope, its parent scope and related imported namespaces and (4) add, overwrite and retrieve the type of an element (e.g. type associated with a variable name) from the current scope, its parent/child scopes, as well as imported namespaces. Without these functionalities, the deduction of types would require continuous redundant model navigations. In our previous work, we did not provide this kind of support.

The generation process is fully automated, and, fed with a valid (UML-)ALF model, it generates valid full-fledged C++. The transformation has been tested and evaluated to assess its applicability to complex models of industrial size also in terms of performance (e.g. scalability).

## Translational execution of ALF

In this section, we present our solution showing the transformation process, its steps and the involved artefacts. The structure of the implementation in terms of Java (Xtend) packages resembles the structure of the ALF specification [36] describing the ALF elements that they translate. This can help for investigating the transformation code. Moreover, additional packages, such as those handling type deduction and the ones taking care of memory management, are separated and leveraged through a set of APIs by the translation packages. This is particularly useful in case additional or alternative mechanisms for, e.g. memory management need to be introduced, or when another similar target language is considered (e.g. Java). Note that the entire transformation process is defined in terms of model transformations. By exploiting the implementation of ALF in terms of metamodelling concepts in Papyrus, the process does not need any parsing activity and operates on the ALF code in terms of its model representation. The transformation process takes into account two possible design scenarios:

**Scenario 1 –**: structural and behavioural details are both given in terms of an ALF model, through units and blocks (sequences of statements), respectively;
**Scenario 2 –**: the software system is modelled using UML (or UML profiles) for defining structural elements. Fine-grained behaviours are defined through UML opaque behaviours with bodies programmed with ALF. In this scenario, legacy behaviours can be defined in terms of other languages.

In the following sections, we describe how the translation of structural and behavioural concepts is carried out in each scenario. Since mechanisms for type deduction and generation of memory management code are exploited by the translation, we describe them first.

### Type deduction

**Fig. 1:**
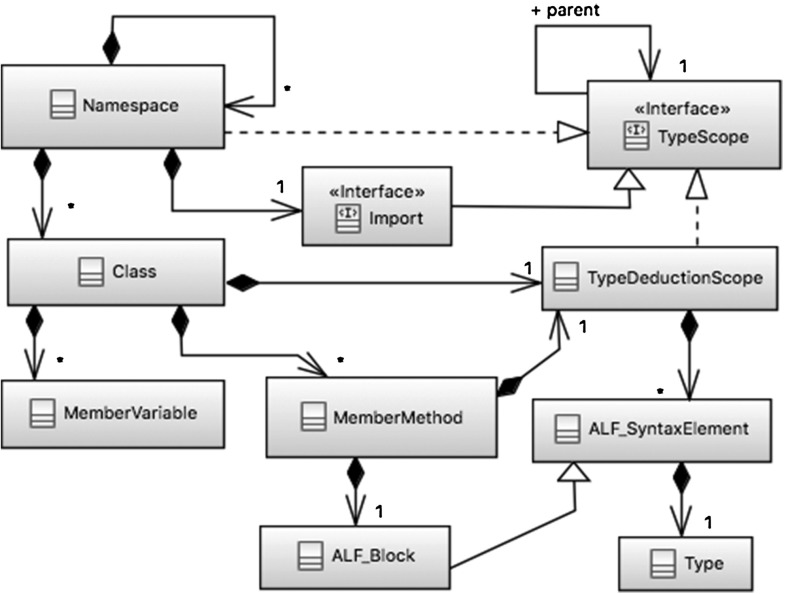
Graphical representation of TypeDeduction

For type deduction purposes, we defined a structure, called TypeDeduction, in terms of Java objects to be naturally compliant to Xtend and thereby directly exploitable by the transformation process. We use TypeDeduction for defining scopes (e.g. a method scope) and variables to them (e.g. a loop variable) and effectively deducing types during the translation of both structural and behavioural modelling elements. In Fig. 1, we depict a graphical representation of TypeDeduction to help the reader to grasp the interdependencies among its constituents.

The core object of TypeDeduction is the TypeScope interface, which defines a set of methods for supporting type deduction. More specifically, TypeScope makes it possible to create and search type scopes for storing and retrieving information about, for example, types returned by expressions, class types, variable definitions and types in the scope of a class or a namespace; specific APIs have been defined for this purpose. Through them, a realisation of TypeScope can search in the tree-based type hierarchy until the specific variable or object is found. Through TypeScope, it is also possible to derive the final type of a fully qualified name as well as of the tokens composing it. An example is given in Sect. 6.

A realisation of TypeScope points always to its direct parent scope through the reference parent; this is used for the scopes to reflect the structural hierarchy from namespaces down to methods and vice versa. TypeScope is realised by TypeDeduction’s main object, Namespace, which corresponds to an ALF package or namespace. Namespace can have a list of Import interfaces, which correspond to ALF import elements. Import extends TypeScope and provides methods for searching the entire imported element scope. Additionally, Namespace can own a list of Class objects corresponding to ALF classes. In turn, Class can have a set of MemberVariable and MemberMethod elements, corresponding to ALF class attributes and operations, respectively. MemberMethod owns always an ALF_Block object, which contains the unmodified body of the related method defined in ALF.

TypeScope is realised even by TypeDeductionScope, which is exploited by Class and MemberMethod to manage their respective scopes and when deducing types within an ALF Block. TypeDeductionScope builds the scope of a specific class or method in terms of the set of ALF syntax elements^7^ (ALF_SyntaxElement) and their types (Type).

The core functionalities provided by TypeScope in terms of APIs can be summarised as follows:

- Scope hierarchy management: scopes are built following their hierarchical structure in the ALF blocks, and APIs are provided for creating, editing and retrieving this information. An example of such is the retrieval of the parent or child scope of the current scope;
- Sub-scope management: sub-scopes associated with an ALF element can be created, overwritten and retrieved;
- Name declarations check: already declared names can be sought within current scope, its parent scope and related imported namespaces;
- Element type management: the type of an element (e.g. type associated with a variable name) can be added, overwritten and retrieved from the current scope, its parent/child scopes, as well as imported namespaces.

Note that TypeDeduction is completely transparent to the developer who simply runs the transformation process and obtains the automatically generated C++ code related to the design model. Moreover, TypeDeduction does not represent an intermediate model or representation between ALF and C++, meaning that the possibility to back-propagate information provided by a compiler for, for example, model-level debugging at model level is not jeopardised.

### Memory management through smart pointers

Due to the platform-independent nature of ALF, an issue to be taken care of when translating it towards a specific platform or programming language is, among others, the management of memory. ALF does not enforce a specific memory management mechanisms, providing the possibility to create and destroy objects explicitly, or to constrain an object’s life cycle to the so-called execution locus” (in C++ it would look like a sort of global object) unless they are not explicitly destroyed. Moreover, even if all the links to an object are explicitly destroyed, in ALF those objects can be retrieved anytime using class extent expressions; in C++, these object would become “unreachable” objects and thereby produce memory leaks. Since there was not a univocal way to bridge memory management mechanisms in ALF and C++, we had to decide upon one. Our goal was to generate C++ code free from memory overflows/leaks for it to be runnable on a large spectrum of systems, including safety-critical ones where memory-related issues are not tolerable [40]. Moreover, since we aimed at providing a solution for industrial usage too, we were restricted to memory management mechanisms for C++ included in the standard specification. When seeking a solution, we considered three main aspects: propensity of generated code towards memory overflows/leaks, complexity of the solution at transformation level and performance of the generated code. We identified three suitable possibilities to solve the memory issue:

- Allocate basic typed variables on the stack and more complex objects on the heap. This solution would result in decent code performance, and it would be fairly easy to implement. The issue with it resides in the fact that it does not ensure prevention from stack overflows since allocations of basic typed variables may require more space than available on the call stack.
- Allocate everything on the heap through *smart pointers*. This solution would give good code performance and would not be extremely difficult to implement. Moreover, it would prevent from stack overflows and memory leaks, since none of the objects are allocated on it.
- Perform a smart allocation based, for example, on the scope of use and the size of the objects. On one hand, this solution would provide the best code performance and prevent from stack overflows. On the other hand, it would be very complex to implement and maintain since it would require (i) an analytical engine to determine object-specific allocations and (ii) a way to automatically generate destructors to handle memory deallocation of user-defined objects.

Since our main focus was to provide a sound solution for memory management that was not too costly to maintain and complement with alternative solutions, we decided to exploit allocation on the heap through *smart pointers*. This kind of pointer defines a reference counter for each allocated object. Once the reference counter hits zero, the related object is released from memory. On one hand, this solution might be costly when dealing with frequent acquisition-release cycles of time-demanding objects. On the other hand, other memory management mechanisms may cause pausing of the whole program execution for the memory to be examined, replaced or freed.

Smart pointers are used to manage objects of non-primitive type (i.e. classes and their instances) in the following way. A non-primitive type T is wrapped as **shared_ptr**< T>, which represents a smart pointer to an object of type T. When instantiating the object (i.e. class instance), the construct **make_shared**<T> is exploited to initialise the smart pointer referencing to the specific object and taking care of memory management.

There are two issues of using smart pointers that can be seen as disadvantages: standardisation and performance [34]. With C++ 11, smart pointers became part of the standard, which is in itself good, but their exploitation can be sensitive in restricted domains, where libraries and frameworks need specific approval. When it comes to performance, smart pointers add two counting variables to manage the memory needed by an object. This represents a tiny overhead in memory usage, usually outplayed by the security that is provided by smart pointers; very few modern embedded systems would not be able to afford such a small overhead. In case of hard real-time applications whose criticality resides in their timeliness, a deeper analysis should be conducted to assess how smart pointers may affect deterioration from a timing perspective.

### Translation of structure

In scenario 1, ALF is used to describe both structure and behaviours of the system under development. The transformation process navigates the ALF structural description and translates ALF units, that is to say declarations of namespaces, packages, classes, properties, methods^8^, to C++ code, and at the same time it creates their scopes by means of TypeDeduction for efficient type deduction when translating ALF behaviours represented by ALF blocks. More specifically, TypeDeduction will be used to deduce types of variables and expressions for, for example, selection of the right member access operator and insertion of smart pointer constructs.

In scenario 2, the structural description is defined using UML and transformed by specific transformations (not part of this contribution). In this scenario, the transformation process only takes care of translating ALF behaviours as described in the next section. Nevertheless, the structural definition in terms of UML is exploited by the transformation for type deduction reasons (creating a TypeDeduction similar to scenario 1) since it gives behaviours the context needed for the transformation to correctly translate them.

### Translation of behaviours

When producing structural C++, either by our transformation process (scenario 1) or by an external code generator (scenario 2), for each UML/ALF operation our code generator checks whether there is a fine-grained behaviour defined in terms of an ALF block. If yes, the generator can perform either of the following three actions depending on how the opaque behaviour is defined:

**Action 1 –**: Behaviour defined in C++ (scenario 2): the generator does not manipulate the action code, but it rather copies it to the corresponding method implementation in the resulting .cpp file;
**Action 2 –**: Behaviour defined in ALF (both scenarios): the generator triggers the model-to-text transformation implementing the translation from ALF to C++ and puts the resulting C++ code in the corresponding method implementation in the .cpp file. Within ALF code, the modeller can define in-line code snippets in other programming languages (as described later in next sections). If the in-line code is written in the language targeted by the transformation, then it is copied to the resulting code, while ignored otherwise;
**Action 3 –**: Behaviour defined in other languages (scenario 2): the generator does not take any specific action. This particular case could be handled by triggering an ad hoc transformation for the specific language (if available) as in action 2, or by performing an action similarly to 1, in case of deployment of different functions (and classes) to different hardware nodes running different target languages.

The transformation process is able to navigate the model, identify ALF blocks and translate them directly to C++. For several reasons, among which correctly generating access to members in C++ and efficiently deducing the type of variables and expressions, type deduction mechanisms are exploited on-the-fly as part of the transformation.

In scenario 2, problems can arise if naming conventions across legacy C++ action code and the C++ code generated by our translator are not aligned; issues can even arise if memory management is handled in different (incompatible) ways by C++ action code (hard-coded memory management) and our code generation process. These issues can be mitigated by adapting memory management mechanisms as well as naming conventions that are considered in the transformation itself. That is to say, in order to bridge differences in naming conventions between the code generated by third-party generators to interact with the one generated by our generator, appropriate APIs (not provided but our transformation) for marshalling/unmarshalling of labels. The effort of providing such an API depends on the level of misalignment in naming conventions and is hard to foresee.

In our solution, since we exploited an open-source code generator for the UML structural portions provided by the author in his previous research work [11], we did not need to provide glue code. When it comes to memory management, an additional reason for which we opted for C+11 standard smart pointers is that they are compatible with other standard memory management mechanism. That is to say, C++ code generated by our translator can interact with legacy C++ exploiting other standard memory management mechanisms.

## Mapping ALF concepts to C++

Our solution provides a novel (first of its kind) translation of the ALF syntactical minimum conformance to C++ except for the following. LinkOperationExpression, used in ALF to create or destroy the links of an association, is not included since associations are not part of the minimum conformance. BitStringUnaryExpression, used in ALF for unary operations on the type BitString, is not included since the type BitString is not conceived in C++. ClassExtentExpression, used in ALF to obtain the objects in the extent of a class, is not included since it is not possible to search all instances of a particular class in C++. Sending instances of a signal in FeatureInvocationExpression is not supported since signals are not part of the minimum conformance. Moreover, it provides the translation of a subset of ALF units, not included in the minimum conformance, for allowing the modeller to define an application using ALF only. In the following, we provide the technology-agnostic description of the supported mappings between ALF abstract syntax elements and corresponding C++ concepts reflecting the order in which ALF syntax elements are described in the official ALF specification (i.e. expressions, statements, units). Since exemplifying all the possible cases of use each syntax element is not possible (they are infinite), we aim at providing a set of representative examples for the reader to be able to reproduce the mappings with the transformation technology of her choice.

Note that, whenever the type of an element (e.g. qualified name, return values) influences the translation from ALF to C++, we exploit our type deductions mechanism for identifying types; in the next sections we highlight the most interesting cases. However, alternative ways to deduce types could be exploited and the mappings described in the following sections are not dependent on the specific deduction mechanism.

### Expressions

Expressions are behavioural units that evaluate to collections of values. In this section, we describe the mappings between the covered types of expression from ALF to C++.

#### Qualified names

QualifiedName is used to identify a UML named element, which may or not be a member of one or more namespaces. To avoid unpredictable C++ code, we did not cover the PotentiallyAmbiguousQualifiedName concept. The remaining concepts are mapped as follows. A qualified name is constituted of non-empty set of bindings, either NameBinding or PositionalTemplateBinding (or a combination of the two). Bindings are separated by colons (‘::’), in case of ColonQualifiedName, or dots (‘.’), in case of DotQualifiedName.

Separation in terms of colons or dots is not univocal in C++ since it depends on the types of the objects represented by NameBinding. More specifically, if the preceding NameBinding represents a class object, colons and dots are mapped to C++’s arrow operator ‘->’ (Case 1, 2, 4); if it represents a property of primitive type, colons or dots are kept in C++ too (Case 3). Regarding PositionalTemplateBinding, since we use smart pointers, the translation is done by wrapping the most internal NameBinding in the **shared_ptr**<T> construct if the name represents a non-primitive type T (Case 5).

The possibility to distinguish among the various cases is given by our type deduction mechanism. More specifically, before translating QualifiedName, we navigates all the bindings composing it and identify the type of each of them. On one hand, in Case 2, type deduction identifies that classA is declared in a parent scope as an instance object of class ClassA; for this reason, the dot ‘.’ operator in ALF is replaced by the arrow ‘->’ operator in C++ (the same applies to Case 1, 4). On the other hand, in Case 3, property is defined in a parent scope as integer, and thereby the dot ‘.’ operator is kept in the resulting C++. In Case 5, PositionalTemplateBinding is done on ClassA and ClassB, which are both identified as class types in their parent scope by the deduction mechanism; the translation is done by wrapping them in the **shared_ptr** $<>$ construct (Table 1).

**Table 1:** Mapping of QualifiedName

Case	ALF code	C++ code
1	pkg::ClassA::property	pkg::ClassA->property
2	classA.property	classA->property
3	property.toString()	property.toString()
4	classA.op()	classA->op()
5	ClassA ${<}$ClassB${>}$	**shared_ptr** ${<}$pkg::ClassA${<}$ **shared_ptr** ${<}$pkg::ClassB${>}{>}{>}$

#### Literal expressions (Primary expressions)

LiteralExpression is composed of a single primitive literal. Since we aim at providing a translator which provides predictable C++ code, we did not account the primitive UnboundedValueLiteralExpression since there is no standard way to translate it to a safe unbounded type in C++. Note that we do not forbid the use of unsupported ALF concepts at modelling level (e.g. through OCL constraints) since models are not only used for code generation purposes. Nevertheless, warnings are issued when generating code from models containing unsupported ALF concepts.

Concerning the other types, BooleanLiteralExpression, StringLiteralExpression and NaturalLiteralExpression, they have a natural corresponding in C++. For instance, a BooleanLiteralExpression in ALF can either be represented by **true** or **false** values of a boolean (**bool**) in C++.

#### Name expressions (Primary expressions)

This syntax element represents the value denoted by a QualifiedName. The mapping to C++ is given by the corresponding qualified name (see Sect. 5.1.1).

#### ‘This’ expressions and Parenthesized expressions (Primary expressions)

ThisExpression consists of the keyword **this**, and it is translated to the same keyword in C++. ParenthesizedExpression represents an expression contained by parentheses; parentheses are simply reproduced in C++, but the contained expression must be properly translated according depending on the expression type.

#### Property access expressions (Primary expressions)

PropertyAccessExpression is used to access the value of a property owned by the instance of a classifier. The expression is defined in terms of a feature reference, pointing to a target primary expression and to a name of a property of the type of the target primary expression. The translation is done by translating the primary expression according to its specific type and relating it to the name of the property to be accessed. In ALF, primary expression and names (NameBinding) are separated by the dot ‘.’ operator, while in C++ this depends on the type of the object to be accessed; this is solved in the same way as for QualifiedName (see Sect. 5.1.1).

#### Invocation expressions (Primary expressions)

This expression represents an invocation to a behaviour and a Tuple, which represents the arguments for the parameters of the invocation. An invocation can be of the following types: BehaviorInvocationExpression, FeatureInvocationExpression and SuperInvocationExpression. Tuple and invocation types are described in the following four sections.

#### Tuple

A Tuple is a list of expressions that describe the arguments for an invocation. They can be positional or named tuples; we provide a translation for positional tuples, since the current specification of C++ does not provide the concepts needed for representing named tuples. A Tuple is translated by iterating on the list of expressions it represents, singularly translate each of them and concatenate their translation using the comma separator ‘,’. The concatenation is put within the parentheses of the translated InvocationExpression. Single expressions are translated according to their type (Table 2).

**Table 2:** Mapping of Tuple

ALF code	C++ code
(par1, “string”, **new** ClassA(),	(par1, “string”, **make_shared**<pkg::ClassA${>}$(),
classA.op2(), {1,2})	classA-${>}$op2(), {1,2})

#### Behaviour invocation expressions (Primary expressions)

It is the simplest type of invocation, and it is represented by a QualifiedName representing the behaviour to be invoked. The translation follows the same rules as the ones defined for QualifiedName (see Sect. 5.1.1). Note that in order for a behaviour called from a model library to be correctly translated, a C++ library corresponding to the model library should be in place. In case C++ library and model library do not share the same naming convention, a wrapper (external to this code generator) should be provide to bridge the differences.

#### Feature invocation expressions (Primary expressions)

This expression has a feature reference as its target, and thereby translated following the rules defined for PropertyAccessExpression (see Sect. 5.1.5), and a final NameBinding representing an operation call. In Case 1, an operation call on a property of primitive type keeps the same syntax in C++. In Case 2, an operation call on a class object is translated by separating the final NameBinding and the accessed property by the arrow operator ‘->’. Case 3 and 4 represented cascaded feature invocation. In Case 3, the return value of *op()* is of primitive type; thereby, it is accessed by the final NameBinding *op2()* through the dot operator ‘.’. In Case 4, the return value of *op1()* is a class object; thereby, it is accessed by the final NameBinding *op2()* through the arrow operator ‘->’ (Table 3).

**Table 3:** Mapping of FeatureInvocationExpression

Case	ALF code	C++ code
1	property.op()	property.op()
2	classA.op()	classA->op()
3	classA.op().op2()	classA->op().op2()
4	classA.op1().op2()	classA->op1()->op2()

#### Super invocation expressions (Primary expressions)

This invocation is used to invoke an operation of a superclass of the current class. Its syntax is similar to FeatureInvocationExpression, but, instead of a feature reference, it has the keyword **super** as target. The mapping is the same as for FeatureInvocationExpression except for the keyword **super**, which is instead substituted by the QualifiedName of the superclass (Table 4).

**Table 4:** Mapping of SuperInvocationExpression

ALF code	C++ code
**super**.op()	pkg::ClassA->op()

#### Instance creation expressions (Primary expressions)

InstanceCreationExpression represents the creation of a new instance of a class or data type. It consists of the keyword **new** followed by a name (possibly qualified) representing the constructor method and a tuple representing eventual parameters for it. The new instance to be created is wrapped in a smart pointer through the construct **make_shared** ${<}{>}$ (Case 1). In case the constructor is retrieved through a PositionalTemplateBinding, the most internal NameBinding is wrapped in a **shared_ptr**<T ${>}$ if it represents non-primitive type T (Case 2). Through our type deduction mechanism, we identify the type of the instance to be created as a class type and wrap it in the **make_shared** ${<}{>}$ construct for initialising a smart pointer for it (Table 5).

**Table 5:** Mapping for InstanceCreationExpression

Case	ALF code	C++ code
1	**new** ClassA(params)	**make_shared** ${<}$ClassA${>}$(params)
2	**new** ClassA${<}$ClassB${>}$()	**make_shared**<pkg::ClassA<**shared_ptr**<pkg::ClassB${>}{>}{>}$()

#### Sequence construction expressions (Primary expressions)

SequenceConstructionExpression groups values into a sequence of a specified type. It is represented by a list of expressions enclosed in curly braces and preceded by the specific type and the multiplicity brackets. A SequenceConstructionExpression that begins with the keyword **new** indicates an InstanceCreationExpression for which a sequence of values is constructed too (Cases 2 and 3). In Case 1, we can see the construction of a sequence of integers and in Case 2 the construction a new array of strings. The mapping is pretty straightforward, except the fact that arrays are mapped to C++’s ‘**vector**’. A more complex case is depicted in Case 3, where a sequence of class objects is constructed by directly creating a new object of the class as first element of the sequence. In this situation, the NameBinding representing the sequence is wrapped into a **shared_ptr** ${<}{>}$, while the NameBinding representing the new class object is wrapped into a **make_shared** ${<}{>}$.

Through our type deduction mechanism, we can identify class types (Case 3) and wrap them in the **shared_ptr** ${<}{>}$ construct for leveraging smart pointers (Table 6).

**Table 6:** Mapping of SequenceConstructionExpression

Case	ALF code	C++ code
1	Integer[]{1, 2, 3}	**vector**<int${>}$({1, 2, 3})
2	**new** String[]{“a”, “bc”, “df”}	**vector** ${<}$string${>}$({“a”, “bc”, “df”})
3	ClassA[]{**new** ClassA(), null}	**vector**<**shared_ptr**<pkg::ClassA${>}{>}${
		**make_shared**<pkg::ClassA${>}$(), null}

#### Sequence access expressions (Primary expressions)

SequenceAccessExpression is exploited to retrieve the element in a specified position of a sequence. It is composed of two expressions, one identifying the sequence followed by one evaluating to an integer representing the index of the element to be retrieved and enclosed in brackets. The two expressions are transformed individually depending on the expression type. The structure of SequenceAccessExpression coincides in ALF and C++.

#### Increment and decrement expressions

This type of expressions uses the increment operator ‘$++$’ or the decrement operator ‘$--$’ in a prefix (operator before operand) or postfix (operator after operand) form for increasing or decreasing an integer operand represented by either a feature reference (FeatureLeftHandSide) or a qualified name (NameLeftHandSide), and an index expression. If the operand is represented by a feature reference, the translation is done according to what is defined for PropertyAccessExpression (see Sect. 5.1.5), while, if represented by a qualified name, it is done as for QualifiedName (see Sect. 5.1.1). The expression providing the index, if any, is translated depending on the expression type.

#### Boolean unary expressions (Unary expressions)

BooleanUnaryExpression is a unary expression composed of: an operand expression which evaluates to a boolean value and the negation operator ‘!’. Its translation is done by properly translating the expression representing the operand, according to the specific expression type, which is preceded by the negation operator ‘!’.

#### Numeric unary expressions (Unary expressions)

NumericUnaryExpression is a unary expression composed of: an operand expression which evaluates to a boolean value and a numeric operator ‘$+$’ or ‘−’. Its translation is done by properly translating the expression representing the operand, according to the specific expression type, which is preceded by the numeric operator.

#### Cast expressions (Unary expressions)

CastExpression is used to cast an operand expression to the type given by a QualifiedName. The translation is done by translating the operand expression according to the specific expression type and the type according to the rules defined for QualifiedName (see Sect. 5.1.1) (Case 1). In the specific case in which the type to cast to is defined as **any**, the type is meant to be derived dynamically at runtime. In this case, **any** is translated to **auto_cast** (Case 2). Type deduction mechanisms support the translation in distinguishing the two cases. Since cast operations are not safe by definition, it is up to the modeller to ensure that the conversion is safe (Table 7).

**Table 7:** Mapping of CastExpression

ALF code	C++ code
(**any**)classA.property	(**auto_cast**)classA->property

#### Binary expressions

A binary expression is composed of two operand expressions separated by a binary operator. Its translation is done by properly translating the expressions representing the operands, according to the specific expression types, and separating them by the specific binary operator. Type deduction mechanisms are exploited for deriving the type of the operands.

ArithmeticExpression is characterised by an arithmetic operator ($+, -, *, /, \%$). Note that arithmetic operator symbols as well as their associativity and precedence rules coincide in ALF and C++.

**Table 8:** Mapping of ClassificationExpression

Case	ALF code	C++ code
1	classA.classB **instanceof** ClassA	**dynamic_cast**<ClassA$*{>}$(classA->classB) $!=0$
2	classA.classB **hastype** ClassA	**typeid**(ClassA) $==$ **typeid**(classA->classB)

**Table 9:** Mapping of AssignmentExpression

Case	ALF code	C++ code
1	classA.classB $=$ **new** ClassB()	classA->classB $=$ **make_shared**<pkg::ClassB>()
2	classB $=$ **new** ClassB()	classB $=$ **make_shared**<pkg::ClassB>()
3	classB[i] $=$ **new** ClassB()	classB[i−1] $=$ **make_shared**<pkg::ClassB>()
4	i $+=$ classA.prop	i $+=$ classA->prop

ShiftExpression is characterised by a shift operator ($<<$ signed left shift, $>>$ signed right shift, $>>>$ unsigned right shift). While signed left and signed right shift operator symbols as well as their associativity and precedence rules coincide in ALF and C++, unsigned right shift ($>>>$) is not available in the C++ specification; hence, we do not enforce its translation.

RelationalExpression is characterised by a relational operator ($<,>, <=, >=$). Relational operator symbols as well as their precedence rules in ALF and C++ coincide.

ClassificationExpression is a peculiar type of binary expression where, instead of the second operand expression, there is a QualifiedName. ClassificationExpression is used to check the result of the operand expression against a certain type represented by QualifiedName. Operand and type are separated by a classification operator (**instanceof**, **hastype**). The operand expression is translated according to the expression type, while the type according to the mapping for QualifiedName (see Sect. 5.1.1). Since the two operators do not have a direct correspondent in C++, we provide the following mappings. In the case of **instanceof**, the expression checks whether the result of the operand expression has the same dynamic type of the given type represented by QualifiedName or a direct or indirect subclass of it. In order to reproduce this behaviour, we dynamically cast the operand to a pointer representing the type we want to check the operand’s type with through the **dynamic_cast** $<>$ operator, and then we check that the result of the casting is not zero (Case 1). In the case the **hastype**, the expression checks whether the result of the operand expression has the same dynamic type of the given type represented by QualifiedName. In order to reproduce this behaviour, we extract the type identifiers of the operand and the given type using C++’s **typeid**() function and compare them through the equality operator ‘$==$’ (Case 2) (Table 8).

EqualityExpression, LogicalExpression and ConditionalLogicalExpression coincide in ALF and C++.

#### Conditional test expressions

ConditionalTestExpression has three operand expressions. The first represents a boolean, and depending on its value, the expression selects either the second or the third operand as result. The mapping is done by translating the three operand expressions according to their expression type and separate them with the symbols ‘?’, between first (boolean) and second operand, and ‘:’ between second and third operand. Conditional test operator symbol ‘?’ and its associativity and precedence rules coincide in ALF and C++.

#### Assignment expressions

AssignmentExpression represents the assignment of a value represented by a right-hand side expression to a left-hand side which can be either a feature reference (FeatureLeftHandSide) or a qualified name (NameLeftHandSide) and can have an index expression. If the left-hand side is represented by a feature reference (Case 1), the translation is done according to what defined for PropertyAccessExpression (see Sect. 5.1.5), while, if represented by a qualified name (Case 2), it is done as for QualifiedName (see Sect. 5.1.1). The expression providing the index, if any (Case 3), is translated depending on the mapping rules for indexing (see Sect. 5.1.21). A simple assignment is done through the assignment operator ‘$=$’. A compound assignment compounds a binary operator with the assignment operator (Case 4) (Table 9).

**Table 10:** Indexing conversion

Case	ALF code	C++ code
1	classA[2]	classA[1]
2	classA[i]	classA[i−1]

**Table 11:** Mapping of LocalNameDeclaration

**Case**	**ALF code**	**C++ code**
1	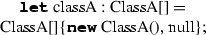	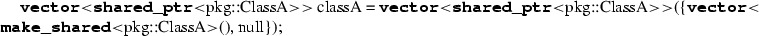
2	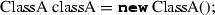	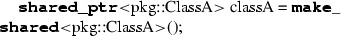
3	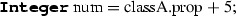	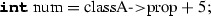

#### Indexing

Since in ALF indexing starts from 1 while in C++ it starts from 0, we need to explicitly make the conversion as follows (Table 10):

- if the index expression is represented by a numeric literal, then we subtract 1 to it (Case 1);
- if the index expression is not a numerical literal, we translate the expression and concatenate ‘$-1$’ to it (Case 2).

**Table 12:** Mapping of IfStatement

**ALF code**	**C++ code**
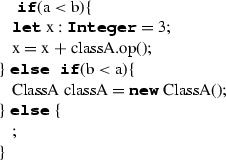	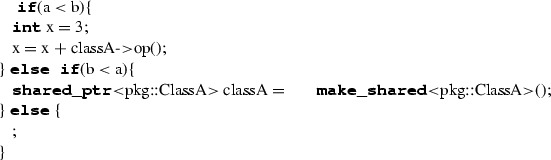

**Table 13:** Mapping of SwitchStatement

**ALF code**	**C++ code**
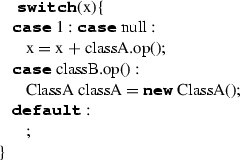	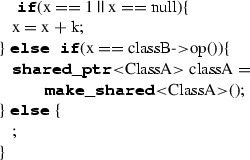

### Statements

Statements are segments of behaviour that, when executed, produce an effect rather than values. A sequence of statements (block) is a list of ALF statements placed side by side in a linear order. These sequences may be included in UML models for specifying behaviours (scenario 2). In this section, we describe the mappings between the covered types of statement from ALF to C++.

#### In-line statements

InLineStatement allows the modeller to embed code in a language other than ALF in an ALF block. No translation is needed in this case since in-line code, if defined in terms of the target language entailed by the transformation, is simply copied as it is in the output code. In our case, we provide support for C++ in-line code, while ignore in-line code defined in other languages.

#### Block statements

BlockStatement represents a block to be executed and can be seen as the container of statements sequence enclosed in curly braces. The concept of block is equally conceived in C++. Before translating a block, the type deduction mechanism creates a sub-scope representing the block’s scope within the current scope.

#### Local name declaration statements

LocalNameDeclaration is a statement which is employed for defining a local name together with its type and initialisation value. It is composed by a name declaration, which can include a multiplicity indicator, and an initialisation expression which can either initialise a sequence, a new instance or be another expression which evaluates to the type of the name to be declared. The syntax of the name declaration has two variants:

- ‘**let** name : Type’, inherited from UML (Case 1);
- ‘Type name’, specific to ALF (Case 2).

Both cases are translated to C++ in the form ‘Type name’. If the initialisation expression defines the initialisation of a new sequence (Case 1), then the expression is translated according to the rules defined for SequenceCreationExpression (see Sect. 5.1.12). In case it defines the initialisation of a new instance (Case 2), the translation follows the rules for InstanceCreationExpression (see Sect. 5.1.11). For the other types of initialisation expressions, the translation is done by the rules defined for the specific expression type (e.g. ArithmeticExpression in Case 3) (Table 11).

#### Expression statements

It is an Expression followed by a semicolon, and it translated according to the rules defined for the specific expression type with a semicolon at the end.

#### If statements

IfStatement represents the conditional execution of a non-empty set of blocks. It is composed by an ordered set of sequential non-final clauses each of which having a condition in terms of a condition expression evaluating to a boolean and a body represented by a block. IfStatement can have a final clause with a block to be executed in case none of the non-final clauses can be executed. Its translation to C++ is done by iterating on the non-final clauses in their order and for each of them transforming the condition expression according to the specific expression type and the statements sequence representing the block (each of the statements will be translated according to the specific type of statement). Sequential non-final clauses are concatenated through the ‘**else**’ keywords both in ALF and in C++. The final clause is translated by translating the related block and concatenating it to the last non-final clause through the keyword ‘**else**’. Before translating IfStatement, the type deduction mechanism creates a sub-scope representing the IfStatement block’s scope within the current scope (Table 12).

**Table 14:** Mapping of ForStatement

**Case**	**ALF code**	**C++ code**
1	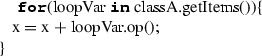	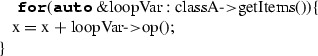
2	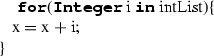	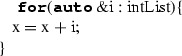
3	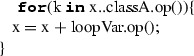	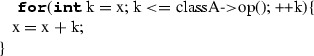

#### Switch statements

SwitchStatement executes one of a set of blocks depending on the value of an expression. The body of the SwitchStatement is made of a list of clauses; each clause consists of a set of case labels and a block. Each case label contains an expression that must evaluate to a single value of a type conforming to the one of the switch expression. As for IfStatement, a switch statement can have a final clause. Case labels are represented by expressions that are dynamically evaluated. In C++, case labels can only be represented by constant expressions; for this reason, we map SwitchStatement to C++’s if-statement. More specifically, we iterate on the set of case labels, which are properly transformed into conditional expressions of **if** and **else if**; the final clause is translated into an **else** without conditional expression. The clauses are translated as follows. An equality condition expression is created to resemble the switch’s cases. Note that multiple cases are translated by creating the related conditional expressions and then using them as operands for a conditional-OR (||) expression. The blocks representing case bodies are translated too. Before translating each clause, the type deduction mechanism creates a sub-scope representing the clause block’s scope within the current scope (Table 13).

#### While and Do statements

WhileStatement and DoStatement are iteration loops which evaluate a condition expression (returning a boolean), and until it becomes false, it executes a block. The translation is done by transforming the condition expression according to its type and statements sequence representing the block. The structure of while-statement and do-statement in ALF and C++ coincide. Before translating WhileStatement or DoStatement, the type deduction mechanism creates a sub-scope representing the specific block’s scope within the current scope.

#### For statements

ForStatement iterates the execution of a block while assigning a loop variable to successive values of a sequence until it reaches the end of the sequence. The translation is done by transforming the loop variable according to its type and then transforming the block. When translating the loop variable, not all the cases have a direct translation to C++. The loop variable can be defined as a name label that assumes values within a sequence returned by an expression (Case 1); the loop variable can be declared explicitly (Case 2). These two cases are mapped to the C++’s range-based for-loop. More specifically, the translation to C++ is done by dynamically typing a reference variable named as the name label through the **auto**. In these two cases, the loop variable is added to the scope of ForStatement through the type deduction mechanism in order to enable its use within the block. Alternatively, the loop variable can be defined as a name label assuming values within a range of integer values with delimiting values represented by two expressions (Case 3). In this case, ForStatement is mapped to a standard for-loop in C++, where the first expression defines the initial value of the loop variable, and the second expression represents the final value. Before translating ForStatement, the type deduction mechanism creates a sub-scope representing the ForStatement block’s scope within the current scope (Table 14).

#### Break statements

BreakStatement is represented by the keyword ‘**break**’ followed by a semicolon, and it is used to stop the execution of an enclosing SwitchStatement, ForStatement, DoStatement and WhileStatement. The translation to C++ is straightforward since the same construct is used in C++ for the same purposes.

#### Return statements

If an operation is expected to return a value, ReturnStatement is used to determine that value and exit the operation. It is composed of the keyword ‘**return**’, which is translated to the same keyword in C++, followed by an expression evaluating to the return value, which is translated according to the specific expression type.

### Units

Units enable the definition of structural elements (mostly in the fUML subset) textually using ALF. In this section, we describe the mappings between the covered types of unit to C++.

#### Namespaces

NamespaceDefinition defines the context for a set of owned members. It can be defined as either a package, through PackageDefinition (see Sect. 5.3.2), or a classifier, through ClassifierDefinition (see Sect. 5.3.3). The visibility of the name of an owned member outside the owner namespace’s scope is defined by a visibility indicator (‘**public**’, ‘**private**’, ‘**protected**’) on the declaration of the owned member. Visibility indicator values coincide in ALF and C++ for properties and methods, but not for classes, which in C++ do not have any visibility indicator.

#### Packages

PackageDefinition is a type of namespace aiming at simply grouping owned members. In our solution, members owned by a package can only be classifiers of type Class. The notion of package in ALF is mapped the C++’s namespace. The translation of PackageDefinition is done by recreating the package structure, but using the keyword ‘**namespace**’. Owned members are translated according to the mappings in the next section. Note that the translation of PackageDefinition produces effects on both the C++ header and implementation files (Table 15).

**Table 15:** Mapping of PackageDefinition

ALF code	C++ header file	C++ impl. file
**package** MyPackage{	**namespace** MyPackage{	**namespace** MyPackage{
}	}	}

**Table 16:** Mapping of ClassDefinition

ALF code	C++ header file
**public class** ClassA{}	**class** ClassA;**class** ClassA{}

**Table 17:** Mapping of PropertyDefinition

ALF code	C++ header file
**private** classB : ClassB[];	**private:** **vector**<**shared_ptr**<ClassB$>>$ classB;

**Table 18:** Mapping of OperationDefinition

**ALF code**	**C++ header file**	**C++ impl. file**
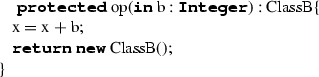	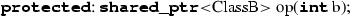	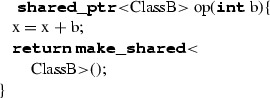

#### Classes (Classifiers)

ClassDefinition represents a classifier whose instances are objects and defines a scope for owned properties and operations. The translation of ClassDefinition is done by recreating the class declaration in C++, that is to say a stub declaration and a forward declaration of the class, and ignoring the visibility indicator. Owned members are translated according to the mappings in the next sections. In this case, the translation of ClassDefinition only affects the C++ header file (Table 16).

#### Properties (Features)

PropertyDefinition is used to define a structural feature of a classifier; in our case, it is used to define attributes of a class. It is composed of a visibility indicator, a type in the form of a QualifiedName, a name in the form of a string and eventually an initialiser expression. The translation is done by reproducing the visibility indicator, transforming the type according to the mapping for QualifiedName (see Sect. 5.1.1) and transforming the initialiser expression, if any, according to the specific expression type. Type deduction is exploited to properly translate the QualifiedName representing the property type; in the example below, the type of classB is identified as an array of ClassB objects which is translated to a **vector** of smart pointers to ClassB (wrapped in the **shared_ptr** ${<}{>}$ construct). The translation of PropertyDefinition only affects the C++ header file (Table 17).

#### Operations (Features)

OperationDefinition represents a behavioural feature of a class. We do not support abstract operations, nor redefinition or overloading. OperationDefinition is composed of a visibility indicator, a name in the form of a string, a set of formal parameters with direction (we only support ‘**in**’ direction), an eventual return parameter and a block. The translation is done by reproducing the visibility indicator and the operation name, transforming the parameters list (including eventual return parameter) and finally transforming the block according to the rules defined for BlockStatement (see Sect. 5.2.2). The translation of parameters is done by considering ‘**in**’ parameters and properly translating their type according to the rules defined for QualifiedName (see Sect. 5.1.1). If the operation does not conceive a return parameter in ALF, the obligatory return parameter in C++ is set to ‘**void**’. This is not the case of constructors, that is to say when OperationDefinition is annotated with *@Create*; as in ALF, a return parameter is not expected in C++ either. Note that the translation of OperationDefinition produces effects on both the C++ header (operation declaration) and implementation (operation implementation) files (Table 18).

## A running example: Self-orienting carrier robot system

The system we exploit for running our solution is represented by a carrier robot self-orienting in a closed environment. The task of this terrestrial robot consists of travelling between checkpoints in a delimited and known environment and simulating item retrieval and delivery. The application is intended to give the robot the ability to orient itself around obstacles of simple shapes; obstacles are created in different places, but within the environment’s delimitations. Similarly, a set of pickup spots and one drop-off spot are created too.

When the robot is initialised, it is placed in the drop-off spot and starts its mission. The robot has then to fetch items from the pickup spots and release them within the drop-off area. The robot moves towards pickup spots and constantly updates its direction until it intersects with the target item. Once the item is picked up, the robot moves towards the drop-off zone and then releases it. The robot has 3 possible directions: forward, right and left. It sorts directions prioritising the one leading to the closest pickup spot, and it moves in a direction as long as it does not intersect with any obstacle. If no direction can be taken, then the robot turns back. The application stops its execution when the robot has picked up and released all the items.

The system is conceived as object-oriented as follows. *Robot* is the main class and exploits two classes, *Vector* and *Hitbox*, for moving in the environment and identifying sensitive spots (its body, pickup and drop-off spots, obstacles), respectively. More specifically, the classes are defined as follows:

- *Vector*: used to define vectorial movements. It contains two properties, *X* and *Y*, defining 2-dimension coordinates, one constructor (*Vector(..)*), and three methods, *vecRotateLeft()*, *vecRotateRight()* and *eq(..)*, which are exploited by the robot to perform movements;
- *Hitbox*: used to describe sensitive spots such as pickup and drop-off areas as well as position and size of obstacles, and the robot’s body size. Positions are identified through *pos* of type *Vector*, while sizes through the properties *height* and *width*. The *Hitbox(..)* method represents the class constructor and the *intersects With(..)* method allows the robot to check whether the movement trajectory intersects an obstacle.
- *Robot*: represents the main class and contains a number of properties and the methods used by the robot to carry out its mission. More specifically, *Robot(..)* is the constructor, *fetch(..)* and *fetchList(..)* are used to retrieve single items and the initial items list, respectively, while the remaining methods allow the robot to move and orient itself in the environment.

In the next section, we use the robot system for showing the translation process.

### Translation of structure

In scenario 1, the structure of the system is supposed to be described in terms of ALF units. In Listing 2, we depict an extract of the description of the self-orienting robot in ALF; the complete ALF description of the robot is available for download (see “Appendix B”). 
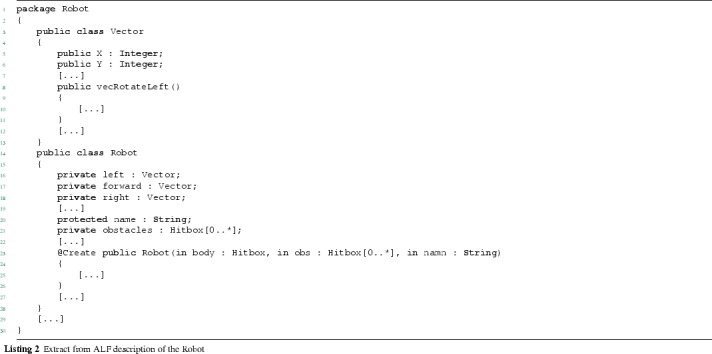
 The translation of the structure produces an instance of the type deduction structure in terms of structural elements and related scopes to be used in the next step for the translation of behaviours. Moreover, it transforms structural elements defined in terms of ALF units to corresponding C++ concepts. These two tasks are intertwined in the transformation.

Considering the ALF code in Listing 2, TypeDeduction would consist of a top Namespace scope, called *Robot*, and representing the related ALF package (line 1 in Listing 2). The ALF classes *Vector* and *Robot* defined in the package are transformed into two Class scope objects, *Vector* and *Robot*, which will be contained by the Namespace. The Class *Vector* scope will in turn contain two MemberVariable objects, *X* and *Y*, corresponding to the properties defined in the Vector ALF class. Moreover, it will contain a MemberMethod object corresponding to the *vecRotateLeft()* operation. The Class *Robot* scope will contain five MemberVariable objects, *left*, *forward*, *right*, *name*, *obstacles* and one MemberMethod object, *Robot*. The bodies of methods *vecRotateLeft()* and *Robot(..)* defined in ALF are stored as ALF_Block objects as part of their corresponding MemberMethod (i.e. *vecRotateLeft()* and *Robot(..)*, respectively) scope, waiting to be transformed in the next transformation step. Type scopes are created in cascade following the hierarchical structure. For instance, Class *Vector* scope will contain the types of the MemberVariable objects, *X* and *Y*, and the scope of the MemberMethod *vecRotateLeft()*. The type scope implementation of *vecRotateLeft()* will have a reference to the type scope of its parent, *Vector*.

The translation of units starts from the PackageDefinition **package** *Robot* which is mapped to the C++’s **namespace** *Robot*. PackageDefinition is translated by recreating the package structure, where owned members are translated according to their type. The translation of PackageDefinition produces the same effect on both the C++ header and implementation (Listing 3) files. 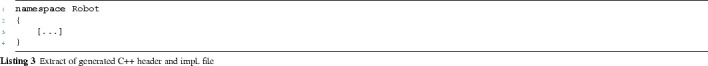


Members of the package are ClassDefinition **public class** *Vector* and ClassDefinition **public class** *Robot*. They are translated to C++’s classes ignoring the visibility indicator. The translation of ClassDefinition only affects the C++ header file (Listing 4). 
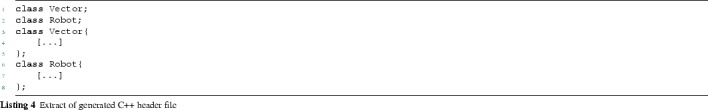


When translating a ClassDefinition object, its owned members are translated too. Let us consider two of the members owned by ClassDefinition **public class** *Robot* starting from PropertyDefinition **private** *obstacles*. The visibility indicator is reproduced as well as the property name. The type of *obstacles* is set to be an array of instances of class *Hitbox* (not shown in Listing 2). The type *Hitbox* is sought in the type deduction structure and found to be a class type, thus needing wrapping as smart pointer through the **shared_ptr** $<>$ construct. Moreover, since *obstacles* is an array, the smart pointer is in turn wrapped in the **vector** $<>$ construct. The translation of PropertyDefinition only affects the C++ header file (Listing 5). 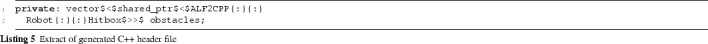 Let us now consider OperationDefinition **public** *Robot(..)*. The translation is done by reproducing the visibility indicator and the operation name and transforming the parameters list; the translation of the block is not part of the structural translation. Regarding translation of parameters, let us consider ‘**in**’ *obs*. The type of *obs* is set to be an array of instances of class *Hitbox*. The type *Hitbox* is sought in the type deduction structure and found to be a class type and is thus wrapped in the **shared_ptr** $<>$ construct. Moreover, since *obs* is an array, the smart pointer is in turn wrapped in the **vector** $<>$ construct. Since OperationDefinition *Robot(..)* is annotated with *@Create*, it is a constructor and thereby a return parameter is not expected in C++. The translation of OperationDefinition *Robot(..)* produces effects on both the C++ header (Listing 6) and implementation (Listing 7) files.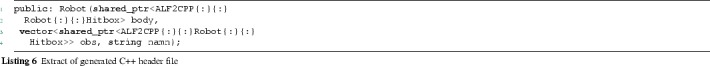 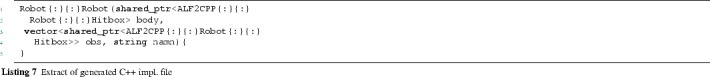 In scenario 2, the structure of the system is defined in terms of a UML class diagram (see “Appendix A”). As aforementioned, in this scenario the transformation does not perform any structural translation, but only builds the type deduction structure reflecting the UML class diagram in order to enable the translation of behaviours. The type deduction structure which is generated recalls the one shown for scenario 1.

### Translation of behaviours

In both scenarios 1 and 2, the behaviours are defined in terms of ALF blocks describing operation bodies. Let us consider the ALF code in Listing 8, which represents a portion of the *updateDirection(..)* operation owned by class *Robot*. The OperationDefinition representing the ALF unit for *updateDirection(..)* is translated similarly to what is shown in the previous section. The block representing the operation’s body is translated as follows. 
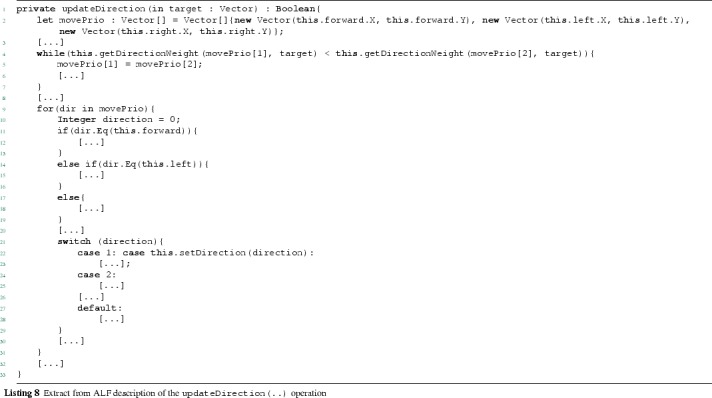
 The first statement we encounter in Listing 8 (line 2) is LocalNameDeclaration *movePrio*. The translation is done following the standard C++ form ‘Type name’ (Listing 9). The type of *movePrio* is set to be an array of instances of class *Vector*. The type *Vector* is sought in the type deduction structure and found to be a class type and is thus wrapped in the **shared_ptr** $<>$ construct. Moreover, since *movePrio* is an array, the smart pointer is in turn wrapped in the **vector** $<>$ construct. The initialisation expression defines the construction of a sequence of objects of type *Vector*. Its translation is done by reconstructing the sequence in C++. The elements in the sequence are represented by ALF expressions of type InstanceCreationExpression, that is to say new instances of type *Vector*. Since *Vector* is a class type, the transformation wraps it in the **make_shared** $<>$ construct for initialising a smart pointer for it. Parameters of InstanceCreationExpression objects are represented by PropertyAccessExpression elements. Let us consider **this** *.forward.X*. Since **this** represents an object of type *Robot*, the access to property *forward* is done in C++ through the arrow ‘->’ operator; since *forward* is of type *Vector*, even the access of property *X* is done through the arrow ‘->’ operator. The translation of the other parameters is achieved in the same way. 
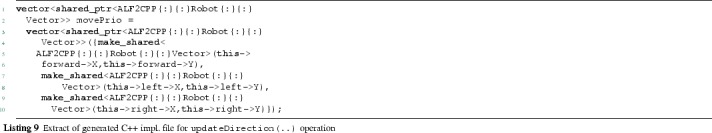
Afterwards, we encounter a WhileStatement (line 4 in Listing 8). Before translating WhileStatement, the type deduction mechanism creates a sub-scope representing the WhileStatement block’s scope within the current scope. The structure of a while-statement in C++ resembles the one in ALF; therefore, the transformation reproduces it. Then, it transforms the condition expression according to its type and the statements sequence representing the block (Listing 10). The condition expression is of type RelationalExpression having two FeatureInvocationExpression as operands and ‘<’ as relational binary operator. For translating RelationalExpression, the transformation needs to translate the two operands, while the operator remains the same. Let us consider the first operand, FeatureInvocationExpression **this** *.getDirection(movePrio[1], target)*. Since it represents an operation call on a class object (**this**, of type *Robot*), it is translated by separating the final NameBinding, *getDirectionWeight(movePrio[1],target)*, and the accessed property, **this**, by the arrow operator ‘->’. The translation of the second operand is done in the same way.

Regarding the block representing the body of WhileStatement, let us consider the first statement, being the ExpressionStatement *movePrio[1] = movePrio[2];* representing an AssignmentExpression (line 5 in Listing 8). Both left- and right-hand sides are represented by indexed feature references. The interesting thing to notice here is the difference in indexing between ALF and C++. Since in ALF indexing starts from 1 while in C++ it starts from 0, and the index expression is represented by a numeric literal, then we subtract 1 to it; the resulting assignment in C++ is *movePrio[0] = movePrio[1];*. 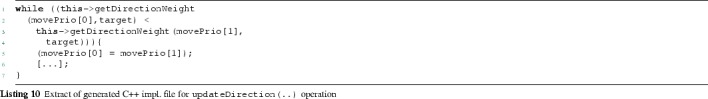 The next statement is a ForStatement (line 9 in Listing 8). Before translating ForStatement, the type deduction mechanism creates a sub-scope representing the ForStatement block’s scope within the current scope. The loop variable is defined a name label (*dir*) that assumes values within a sequence represented by the QualifiedName *movePrio*. The C++’s range-based for-loop is used in this case (Listing 11). More specifically, the transformation creates a reference to the variable loop (*&dir*) and type it as **auto** (for dynamic typing). The variable is also added to sub-scope representing the ForStatement block’s scope in order to enable its use in the block. At this point, the transformation translates the block of the ForStatement. The first statement is represented by LocalNameDeclaration *direction* (line 10 in Listing 8). The translation is done following the standard C++ form ‘Type name’. The type of *direction* is set to be a primitive **Integer**, thus corresponding to **int** in C++. The initialisation expression defines a simple AssignmentExpression to a NaturalLiteralExpression ($=$ *0*), which is reproduced in C++.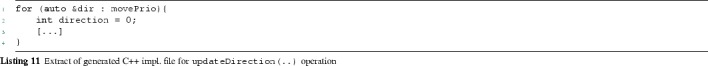 As part of the previous ForStatement’s block, the transformation encounters an IfStatement (line 11 in Listing 8). Before translating IfStatement, the type deduction mechanism creates a sub-scope representing the IfStatement block’s scope within the current scope. The structure of an if-statement in C++ resembles the one in ALF; therefore, the transformation reproduces it by properly translating its clauses (Listing 12). More specifically, the transformation iterates on the non-final clauses in their order, and for each of them, it transforms the condition expression according to the specific expression type and the statements sequence representing the block. Let us consider the first non-final clause **if** *(dir.Eq(* **this** *.forward))*. The condition expression is represented by a FeatureInvocationExpression. Since it represents an operation call on a class object (*dir*, of type *Vector*), it is translated by separating the final NameBinding, *Eq(* **this** *.forward))*, and the accessed property *dir* by the arrow operator ‘->’. Then, the clause block is translated. The second non-final clause is translated in the same way. The final clause is transformed by translating the related block and concatenating it to the last non-final clause through the keyword ‘**else**’. 
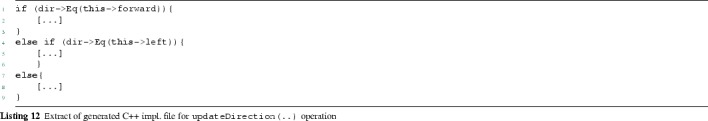
 In the ForStatement’s block, the transformation encounters a SwitchStatement too. The translation is done by iterating on the set of case labels and transform them into condition expressions of ‘**if**’ and ‘**else if**’; the final clause is translated into an **else** without condition expression (Listing 13). Before translating each clause, the type deduction mechanism creates a sub-scope representing the clause block’s scope within the current scope (as for the IfStatement). The blocks representing case bodies are translated too. Let us consider the first (multiple) case label, ‘**case** *1:* **case** **this** *.setDirection(direction):*’. The second part of the combined case label is represented by a non-constant expression; this is not allowed in the switch statement in C++, and that is the reason why we map SwitchStatement to C++’s if-statement. The translation of the multiple case label is done by creating the related condition expressions and then using them as operands for a conditional-OR ‘||’ expression.
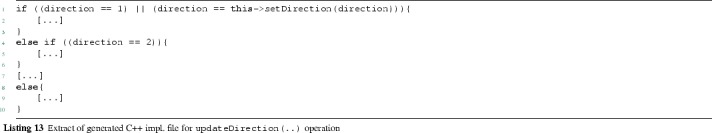


## Validation

The outcome of the transformation mappings between ALF and C++ has been syntactically examined through transformation unit testing [50] exploiting the JUnit facilities provided in Eclipse. More specifically, for testing the translation of ALF syntax elements to C++ we defined a set of test cases consisting of a pair <ALF_file, C++_file>, where ALF_file represented the ALF statements we wanted to test and C++_file the corresponding code (manually written) we expected the transformation to produce as valid translation of ALF. The test cases were run by the JUnit engine that, for each pair <ALF_file, C++_file>, runs the transformation on ALF_file to generate corresponding C++ code. Actual (generated C++) and expected (C++_file) results were parsed and compared to check whether they indeed matched. To enhance the accuracy of the comparison, actual and expected C++ code snippets were reformatted by removing new lines, spaces and tabs. On one hand, this mechanism helped us uncover several bugs in the transformation. On the other hand, it could not prevent the possibility of false positives. Since this testing approach is sensitive to variable naming, we ensured that manually written expected C++ followed the same naming conventions as the translation process.

A similar mechanism was used to test type deduction. In this case, test cases were written as a pair <ALF_file, Scope_file>, where Scope_file represented the expected (manually written) result in the form of a scope file containing scopes, variable definitions and their deduced types. When the test cases were run, scope hierarchy, variables and related deduced types from ALF_file were generated in a resulting scope file. Actual and expected scope files were parsed and compared to check whether they matched. Individual test cases have been defined and run for each of the translatable ALF syntax elements. Totally, 77 test cases distributed across 175 files have been defined and successfully run. Combined test cases were defined and run for testing the ability of the transformation to successfully translate complex combinations of syntax elements (i.e. complex ALF blocks).

The solution we provide in this paper has been validated exploiting several applications. Among them, the self-orienting carrier robot example was employed in the paper for showing the translation process considering its suitable balance between simplicity and veracity. Nevertheless, for a deeper validation of the functional correctness of the generated C++ code as well as an evaluation of the scalability of the transformation process^9^, we exploited industrial models of various verbosity. More specifically, we leveraged the Asynchronous Transfer Mode (ATM) Adaptation Layer 2 (AAL2) subsystem, originally developed to adapt voice for transmission over ATM and currently used in telecommunications as part of connectivity platform systems. The complete AAL2 subsystem is composed by several hundred thousands of component instances, multiple levels of hierarchical composition of components, and several hundred thousands lines of action code defined in ALF. The AAL2 subsystem models, defined within Ericsson Nikola Tesla in Zagreb (Croatia) under the supervision of Ericsson AB in Kista (Sweden), on which the solution was applied, consisted of a maximum of 3000 component instances and 15000 port instances decomposed in a maximum of 10 hierarchical composition levels [27]. The generated C++ code could successfully be compiled and run on an Ericsson’s node in simulated environment (at Ericsson Nikola Tesla). The functional correctness of the generated code was assessed by observing and reporting from the code execution through monitoring and logging routines, respectively; generated code was manually instrumented to produce a log with data and control flows for checking functional correctness. When it comes to the user experience of using ALF instead of C++ for defining behaviours in the AAL2 UML models, industrial modellers found it very intuitive thanks to its Java-like syntax and appreciated the naturalness by which it seamlessly integrates with UML. Clearly some domain-specific C++ constructs could not be used and a workaround using ALF concepts had to be found; this was balanced out by the fact that having to use ALF to remodel the system made them uncover potential improvements to the behavioural code.

**Table 19:** Transformation performance

# ALF LoC	# Number runs	Avg. transform time
130	100	19.3 ms
1015	100	99.5 ms
10455	100	989.2 ms
109335	100	10.9785 s

In Table 19, we show a summary of the results we gathered by running the translation process. The transformation, run on four versions of the AAL2 model from the smallest composed of 130 lines of ALF code to the biggest composed of 109335 lines, was always able to generate full-fledged valid C++. Generated C++ displayed same data and control flows of the legacy code (also generated C++), measured thanks to monitoring routines defined as specific processes extending the kernel of the operating system. Moreover, we computed the average time that the transformation took to complete its task, on a number of runs. As it can be seen in the table, the transformation displays a linear behaviour and completes its task in a reasonable amount of time considering its idiosyncratic intricacy.

## Discussion

The translation process we presented in this paper allows to generate full-fledged C++ from a software system defined in terms of ALF (and UML). Although tested and evaluated, the validity of the transformation does not induce the validity of the generated C++. In other words, the transformation produces code which effectively represents what is defined in the model; if the model is not valid, then the transformation is not able to produce valid C++. Generally, while syntactical correspondence between ALF and generated code can be controlled and ensured by the transformation since the validity of ALF models syntax is ensured by the Papyrus environment with a set of validation features, the same cannot be said for the execution semantic correspondence. We have performed several tests to check that generated C++ resembles the execution semantics of the corresponding ALF model, as specified by the underlying fUML. Even though we did not identify pitfalls in this semantic correspondence, in order to provide more tangible evidences, automated comparisons of execution traces gathered from the execution of ALF and the generated C++ should be performed. Anyhow, it would be hard to prove semantic equivalence [18] between the two languages once and for all, since different implementations of them, executed on different platform configurations, would most likely show variations in their behaviours.

The use of action languages for specifying complex behaviours within UML is not new, several approaches can be found in the literature as described in Sect. 3. A UML-based modelling approach leveraging a programming language for the definition of fine-grained behaviours makes code generation easier since action code is simply copied, as it is, in the resulting code. The main issue is that this habit does not allow the modeller to have full control on the correctness of the modelled behaviours. Additionally, using programming languages for action code, models are bound to a specific platform (or set of platforms) already at functional modelling level. By employing an action language like ALF, action code is empowered with full knowledge of the surrounding model elements [45, 48]. This triggers several benefits, among which simplified model-based analysis, model simulation and consistency checking at modelling level, to mention a few. Regarding the reusability of models, since ALF is not bound to any specific target platform, code generators can target different variations (providing some degree of reusability for code generators too) of one language (e.g. different memory management mechanisms depending on the user’s selection) or different languages, from the same model. This means that ideally from the same UML–ALF model it should be possible to provide generators that produce, e.g. C++, Java or even C, for different platforms and accounting different variations. While in this paper we touched upon a gradual adoption of ALF-compliant modelling by mixing ALF and C++ for the definition of fine-grained behaviours, it should be clear that developers get to enjoy full platform independence only if the sole ALF is used for fine-grained behaviours, with no opaque behaviours defined in other languages (such as C++ in our example).

With the standardisation of ALF, we have noticed an increasing industrial interest in gradually moving towards its adoption. Clearly, this adoption will not be easy nor fast, since the use of programming languages within models is entrenched in those industrial processes exploiting MDE through UML. Providing this translational solution for ALF, we aim at boosting this process by giving the possibility to exploit our transformation as a complement to existing transformations. We believe that using our transformation process as a complement to powerful transformations for structural and deployment aspects represents the most attractive way to exploit it. Suppose that, instead of using UML for structural modelling, we would like to employ a specific UML profile and a specific transformation generating structural C++ code from it. Our solution for translating ALF to C++ would not be affected by the distinctive differences between UML and its profile and thereby could be employed without major changes; this has been validated by employing the transformation process on models defined by means of the CHESS-ML profile in [7]. Moreover, once a stable version of the C++ code generator for the UML-RT profile within the Papyrus Industrial Consortium initiative will be available, we will run an experiment to assess its interplay with our code generator for ALF.

Overall, code generators for UML-based MDE already leveraging transformations from UML (and profiles) with C++ as action language to executable C++ can benefit from reusing legacy components while exploiting a fully model-driven approach designing new components entirely using UML and ALF. An interesting direction that stems from the contribution presented in this work is to look into reverse engineering of C++ code (legacy or generated) to ALF for consistency assurance; existing works in this kind of support for UML can be found in the literature [16].

Our solution is also meant to further stimulate interested developers and companies in adopting MDE and UML. At its birth, UML was mainly regarded as a way to describe purposes, support analysis, design and document since its semantics was more ambiguous and weaker than well-established programming languages. For these reasons, the initial interest of developers and companies in MDE, and specifically in UML, partially dissipated. With its later embodiment and eventually the standardisation of its execution semantics (fUML) and action language (ALF), UML has become a full-fledged implementation quality language [45]. By providing automation in the translational execution of its recently standardised action language, we aim at giving our contribution the power to smooth the way for practitioners in adopting MDE and UML.

Our effort in translational execution is justified by the state of the practice in industry which is pervaded by code generation for many reasons, among which reusability of existing runtime layers and optimised compilation of high-level code (e.g. C++). With that said, to minimise semantic pollution typical of translational approaches and due to the distinctive differences between modelling and programming languages, our longer term goal is to address direct compilation of ALF (and UML). On one hand, doing so, semantic discrepancies and the consequent need of compromises typical of languages translation could be minimised. On the other hand, the way towards an industry-quality compiler for ALF (and UML) is far from unhindered, since abstraction and expressive power of these languages must somehow be tamed. Moreover, already existing domain- or company-specific optimisations for specific programming languages and compilers shall be reproduced too. The latter would also entail the need for certification of the eventual ALF compiler/interpreter in specific domains (e.g. safety-critical applications). Looking at the history of well-established compilers, this would not be a swift task, but its outcome could bring a long list of benefits.

## Conclusion

In this work, we presented a solution for the *translational execution* of ALF towards C++ defined in terms of model-to-text transformations. More specifically, we provided (i) a translation within the syntactical *minimum conformance* (as described in the ALF specification), used for writing textual action language snippets as behaviours within larger UML models, (ii) a translation of a set of ALF units (namespace, package, class (passive), operation, property), (iii) a memory management mechanism based on *smart pointers* and (iv) a type deduction mechanism.

In this paper, we focused on the minimum conformance, as defined in the ALF specification, that includes all the capabilities available in a traditional, procedural programming language. Doing so, we were able to ‘program’ fully fledged UML–ALF models even in industrial settings. Nevertheless, the minimum conformance has a drawback. Constructs that make the language powerful such as the possibility to navigate and filter collections using OCL-like expressions are not available. We have already started looking into possible solutions for the inclusion of OCL-like expressions in our translator [25].

An interesting enhancement of the translation process would be the possibility for the developer to configure the transformation through parameters in order to reach a specific result. An example of this could be the possibility to exploit different mechanisms for memory management and let the developer select the one to use when launching the transformation. The transformation process has been designed in a way that should enable partial reuse of the code generator when targeting another object-oriented language. We have planned to add Java as target language for our code generator. Doing so, we would be able to measure the actual effort needed for such an enhancement. Moreover, we have already started an effort towards direct compilation of UML and ALF, without intermediate translations to programming languages such as C++, to see to which extent we can preserve execution semantics of ALF in the generated executables.

## Figures and Tables

**Fig. 2:**
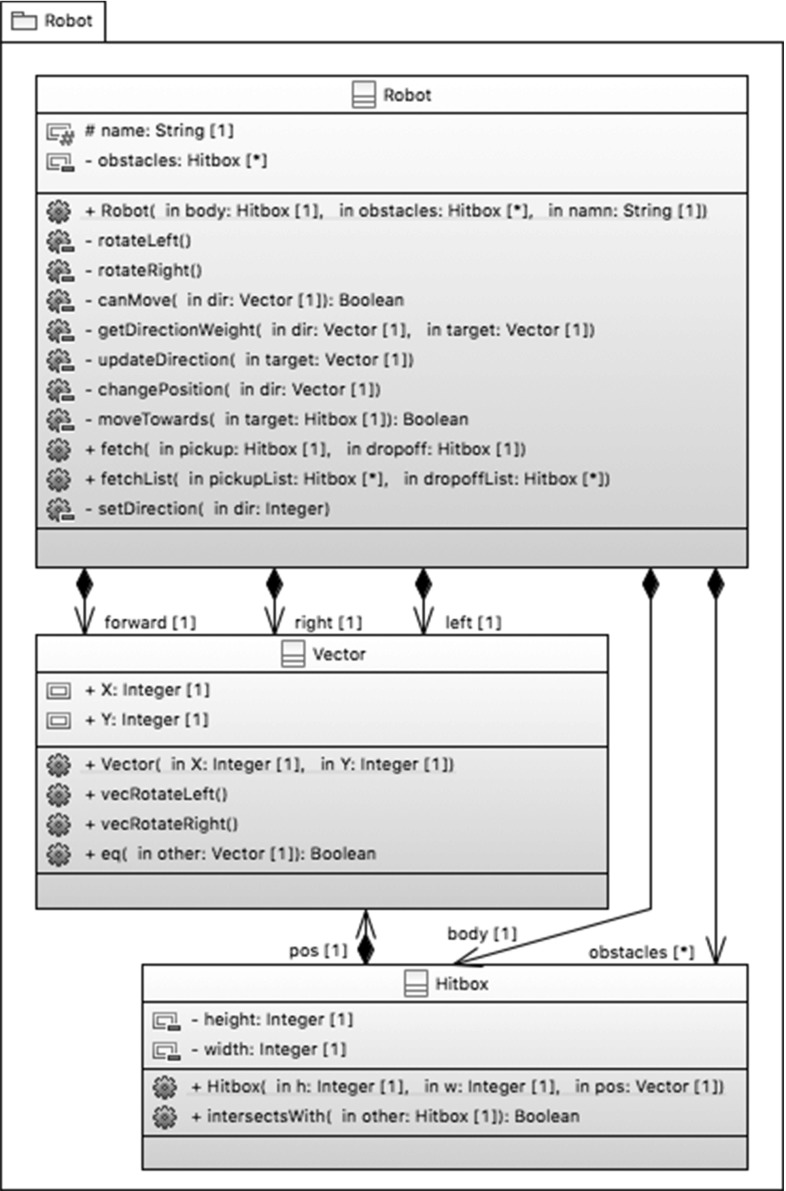
UML class diagram for the self-orienting carrier robot system

**Fig. 3:**
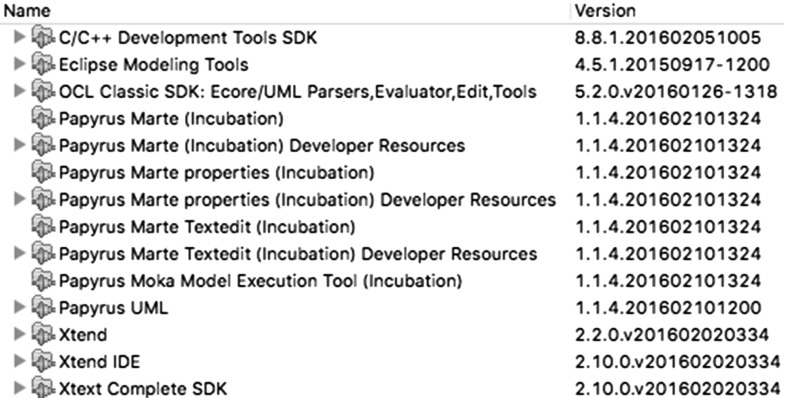
Eclipse installation details

## Appendix A UML class diagram describing the structure of the self-orienting carrier robot system

See Fig. 2.

## Appendix B Guidelines for running the solution

For the interested reader, a working prototype of the solution is available at http://www.mrtc.mdh.se/ALF2CPP/. More specifically:

- Plug-ins implementing the transformation from ALF only to C++ (scenario 1) can be found under http://www.mrtc.mdh.se/ALF2CPP/ALF_ONLY_to_CPP/. The ALF model of the self-orienting carrier robot can be found at http://www.mrtc.mdh.se/ALF2CPP/ALF_ONLY_to_CPP/model.
- Plug-ins implementing the transformation from ALF blocks within a UML model to C++ (scenario 2) can be found under http://www.mrtc.mdh.se/ALF2CPP/ALF_in_UML_to_CPP/. The UML model of the self-orienting carrier robot containing ALF behaviours can be found at http://www.mrtc.mdh.se/ALF2CPP/ALF_in_UML_to_CPP/model.

In order to run the solution, choose the scenario you prefer, download related plug-ins and example model from the specific links given above. The plug-ins should be imported in your Eclipse workspace. Once the plug-ins are imported, run a new Eclipse (runtime) configuration from the one hosting the plug-ins. In the runtime Eclipse instance you can now import the example model. At this point, you can run the transformation as follows:

- Scenario 1 (from Robot.alf to C++): right-click on the robot model Robot.alf and select the action ‘Transform to C++’. In the same project containing Robot.alf, a new folder ‘src-gen’ will be created and will contain the generated C++ files.
- Scenario 2 (from Robot papyrus model to C++): right-click on the Robot.uml (or uml in the Robot papyrus model) and select the action ‘Transform to C++’. In this case, no files are generated, but the translated ALF blocks in C++ are printed out in the Console of the Eclipse instance hosting the plug-ins (not the runtime Eclipse instance where the model is imported).

The installation details of the latest Eclipse configuration on which the solution has been run are shown in Fig. 3.

## Footnotes

1
http://www.uml.org/.

2
https://www.eclipse.org/org/workinggroups/papyrusic_charter.php.

3
https://eclipse.org/xtend/.

4
http://www.omg.org/spec/OCL/2.4/.

5
http://www.scala-lang.org/.

6
http://www.cplusplus.com/reference/memory/shared_ptr/.

7 Note that any node of an ALF syntax tree is referred to as *syntax element*.

8 Note that in the paper we use interchangeably *method*, *operation* and *function* as synonyms.

9 Scalability of the transformation in terms of its execution time was regarded as a core priority by our industrial partners.
